# Coupled Integration of CSAC, MIMU, and GNSS for Improved PNT Performance

**DOI:** 10.3390/s16050682

**Published:** 2016-05-12

**Authors:** Lin Ma, Zheng You, Tianyi Liu, Shuai Shi

**Affiliations:** 1 Department of Precision Instrument, Tsinghua University, Beijing 100084, China; malin10@mails.tsinghua.edu.cn (L.M.); liuty14@mails.tsinghua.edu.cn (T.L.); shi-s11@mails.tsinghua.edu.cn (S.S.); 2 State Key Laboratory of Precision Measurement Technology and Instruments, Tsinghua University, Beijing 100084, China

**Keywords:** integration, CSAC, MIMU, GNSS, precise time aiding, Kalman filter

## Abstract

Positioning, navigation, and timing (PNT) is a strategic key technology widely used in military and civilian applications. Global navigation satellite systems (GNSS) are the most important PNT techniques. However, the vulnerability of GNSS threatens PNT service quality, and integrations with other information are necessary. A chip scale atomic clock (CSAC) provides high-precision frequency and high-accuracy time information in a short time. A micro inertial measurement unit (MIMU) provides a strap-down inertial navigation system (SINS) with rich navigation information, better real-time feed, anti-jamming, and error accumulation. This study explores the coupled integration of CSAC, MIMU, and GNSS to enhance PNT performance. The architecture of coupled integration is designed and degraded when any subsystem fails. A mathematical model for a precise time aiding navigation filter is derived rigorously. The CSAC aids positioning by weighted linear optimization when the visible satellite number is four or larger. By contrast, CSAC converts the GNSS observations to range measurements by “clock coasting” when the visible satellite number is less than four, thereby constraining the error divergence of micro inertial navigation and improving the availability of GNSS signals and the positioning accuracy of the integration. Field vehicle experiments, both in open-sky area and in a harsh environment, show that the integration can improve the positioning probability and accuracy.

## 1. Introduction

Positioning, navigation, and timing (PNT) is important for vehicles, aircrafts, robots, and pedestrians, especially for military use. Global navigation satellite systems (GNSS) based on radio signals and inertial navigation systems (INS) based on Newton’s law are widely used forms of PNT technology. GNSS can provide automatic, continuous, low-cost, global coverage in all-weather with high-precision positioning and timing. However, the satellites are thousands of kilometers away from the ground and the signal transmission power is limited by the solar power collector. Thus, satellite signals arriving at the ground are extremely weak and susceptible to occlusion and interference, which can cause navigation interruption. The poor dynamic performance of GNSS makes it difficult to provide continuous PNT information to high-speed moving carriers [1,2,3,4]. INS measures acceleration and angular velocity by inertial sensors, and calculates position, velocity, and attitude by an inertial navigation algorithm. INS provides autonomous, high refresh rate, good short-term accuracy, and stable navigation information. INS can work in the air, ground, underground, underwater, and indoors. With the development of micro-electromechanical systems (MEMS) technology, the performance of MEMS accelerometers and gyroscopes have been improved, producing more accurate, smaller, cheaper, and more developed devices with time. MEMS inertial measurement units (MIMU) constructed by three axis accelerometers and gyroscopes are used in INS. However, the biggest drawback of INS is error accumulation with time [5,6,7,8].

Integration of navigation systems is an effective way to overcome the drawback of a single navigation method and improve PNT performance. An integrated navigation system combining GNSS and MIMU can provide information regarding position, velocity, altitude, angular velocity, acceleration, and time. A MIMU/GNSS integrated navigation system has the advantages of both satellite and inertial navigation technologies and is able to overcome each system’s individual shortcomings. MIMU/GNSS offers high precision, good reliability, small volume, low dynamic stress sensitivity, and no error accumulation [9,10,11,12,13]. According to the depth of coupling, MIMU/GNSS combination can be divided into three categories: loosely coupled, tightly coupled, and deeply coupled. Zhao [14] and Chang [15] proposed several filter methods for loosely coupled MIMU/GNSS. Tawk [16] and Rabbou [17] proposed several loop aiding architectures for tightly coupled MIMU/GNSS. Ban [18] analyzed how MIMU quality affects deeply coupled MIMU/GNSS. Deeper combined depth usually provides better accuracy and robustness of the system. However, the theory of deeply coupled systems remains unclear and has not been applied.

Atomic clocks provide the most-precise frequency reference for humans. The frequency accuracy of atomic clocks is several orders of magnitude higher than that of crystal oscillators [19]. The chip scale atomic clock (CSAC) was fabricated by USA National Institute of Standard and Technology in 2002 [20]. The size, weight, power, and cost (SWaP+C) of CSAC are considerably better than those of traditional rubidium atomic clocks. CSAC has 120 mW power consumption, 40.6 mm × 35.5 mm × 11.4 mm physical size, and higher than 1.5 × 10^−10^ at 1 s stability (*i.e.*, higher than 5 × 10^−11^ at 10 s stability for the traditional clock [21]). Commercialization of CSAC extends the applications of atomic frequency reference. For PNT applications, CSAC not only improves timing accuracy but can also be treated as a satellite for GNSS. Sturz [22], Van Graas [23], Misra [24], Kline [25], Zhang [26], and Bednarz [27] found that traditional rubidium clocks improve dilution of precision of GNSS and positioning accuracy. Recently, Ma [28] proposed that deeply coupled CSAC and GNSS can improve PNT robustness.

Combining CSAC, MIMU, and GNSS has the advantages of MIMU/GNSS and CSAC/GNSS. With precise time aiding, the integration of CSAC, MIMU, and GNSS cannot be degraded to inertial navigation even when only one satellite is visible. Thus, the GNSS information can be used as fully as possible and the PNT availability can be improved. Research on the integration of CSAC, MIMU, and GNSS has not been reported. Thus, this study explores the coupled integration of CSAC, MIMU, and GNSS for PNT performance. Section 2 discusses the architecture of coupled integration, Section 3 derives mathematical model of precise clock aided integrated navigation, Section 4 describes the field vehicle experiments, and Section 5 presents the conclusions.

## 2. Architecture of Coupled Integration

**Figure 1:**
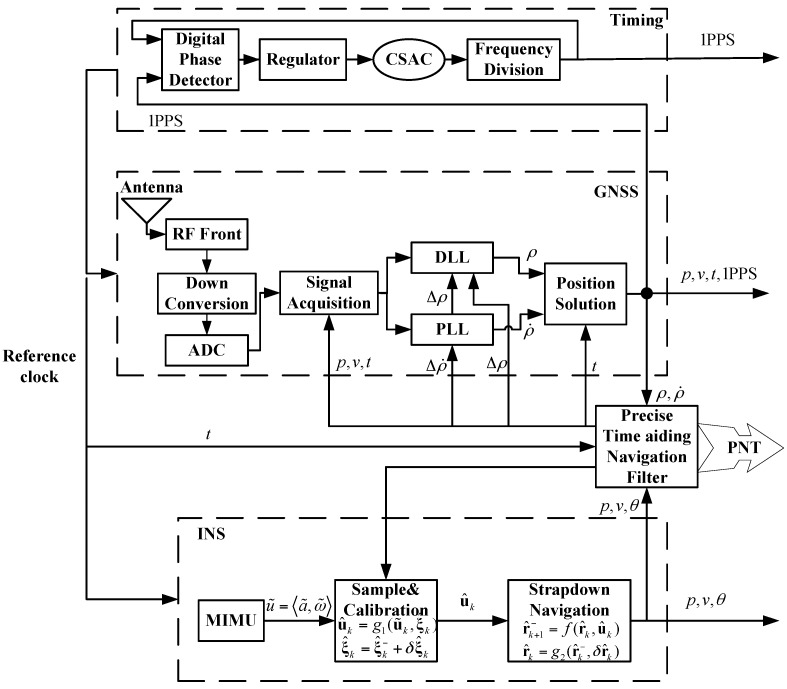
Architecture of chip scale atomic clock (CSAC), MEMS inertial measurement unit (MIMU), and global navigation satellite systems (GNSS) coupled integration. MEMS refers to micro-electromechanical systems.

The architecture of CSAC, MIMU, and GNSS coupled integration is described in Figure 1; it includes four blocks, namely, timing, GNSS, INS, and precise time-aiding navigation filters.

The timing block provides timing information. Usually, when the GNSS constellation geometry is good, one pulse per second (1 PPS) signal is synchronized with Coordinated Universal Time (UTC) with an error of only several nanoseconds. Thus, the start time of CSAC is determined by GNSS. One pulse per second signal enters the timing block and uses a feedback loop to restrain error accumulation caused by “clock coasting” of CSAC. This loop also reduces the noise of 1 PPS signal, and it consists of a digital phase detector, regulator, CSAC, and frequency division. If GNSS cannot provide 1 PPS signal, then this loop degrades to clock coasting mode.

In the INS block, carrier motion can be described as $\tilde{u}=\langle \tilde{a},\tilde{\omega }\rangle$, which includes three-dimensional acceleration and angular rate. MIMU measures the carrier motion. After sampling and calibration, the measurement can be expressed as follows:

(1) $$\begin{array}{l} {\hat{u}}_{k}=g_{1}({\tilde{u}}_{k},{\hat{\xi }}_{k})\\ {\hat{\xi }}_{k}={\hat{\xi }}_{k}^{-}+\delta {\hat{\xi }}_{k} \end{array}$$

Initial calibration parameters of MIMU can be determined by laboratory tests or a fast field calibration method. When the navigation filter works, it provides the parameters.

The strap-down inertial navigation algorithm is

(2) $$\begin{array}{l} {\hat{r}}_{k}^{-}=f({\hat{r}}_{k},{\hat{u}}_{k})\\ {\hat{r}}_{k}=g_{2}({\hat{r}}_{k}^{-},\delta {\hat{r}}_{k}) \end{array}$$

In the GNSS block, the clock signal is provided by the timing block. The antenna, Radio Frequency (RF) front, down conversion, and analog to Digital Converter (ADC) transfer the satellite RF signals to base band signal. Acquisition of base band signal is aided by the position, velocity, and time information from the navigation filter. Δρ and $\Delta \dot{\rho }$ are pseudo range increment and pseudo range rate increment, respectively, which are from the navigation filter. The delay lock loop (DLL) used for pseudo random code tracking is aided by Δρ and phase lock loop (PLL) used for carrier tracking is aided by $\Delta \dot{\rho }$. These aids can improve the dynamic stress tolerance of GNSS.

In the precise time-aiding navigation filter block, precise time, pseudo range, pseudo range rate, and INS output are transmitted to the block. Process and observation models are constructed based on precise time, and the Kalman filter is used to integrate navigation information. The calibrated information is transmitted to sensors and the error parameters are corrected. The PNT information is provided as the integration’s output.

The coupled integration can be degraded because of the failure of subsystems. Table 1 shows degradations of the coupled integration. In the table, “√” or “×” means that the sensor works or fails, respectively. If all of the subsystems work well, then the integration works in coupled integration mode. If CSAC fails, then the integration works in MIMU/GNSS tightly coupled integration. If MIMU fails, then the integration works in CSAC/GNSS coupled integration mode. If GNSS fails, then the integration works in inertial navigation mode.

## 3. Mathematical Model of Precise Time-Aiding Navigation Filter

The precise time-aiding navigation filter is based on the Kalman filter. The integration state model is constructed by inertial sensors and algorithms, and the integration observation model is constructed by GNSS information with precise time aiding. The navigation filter is defined as Λ.

### 3.1. Integration State Model

The state vector X is defined as:

(3) $$X=\left[\begin{array}{ccccccc} \delta R & \delta V & \Phi & \nabla & \varepsilon & \delta SF_{a} & \delta SF_{g} \end{array}\right]$$

where $\delta R=\left[\begin{array}{ccc} \delta \varphi & \delta \lambda & \delta h \end{array}\right]$ is the error of latitude, longitude, and altitude of MEMS INS, $\delta V=\left[\begin{array}{ccc} \delta v_{E} & \delta v_{N} & \delta v_{U} \end{array}\right]$ is the error of east, north, and up velocity, $\Phi =\left[\begin{array}{ccc} \alpha & \beta & \gamma \end{array}\right]$ is the error of pitch, roll, and yaw angles, $\nabla =\left[\begin{array}{ccc} \nabla_{x} & \nabla_{y} & \nabla_{z} \end{array}\right]$ is the three-axis accelerometer bias, $\varepsilon =\left[\begin{array}{ccc} \varepsilon_{x} & \varepsilon_{y} & \varepsilon_{z} \end{array}\right]$ is the three-axis gyroscope bias, $\delta SF_{a}=\left[\begin{array}{ccc} \delta SF_{ax} & \delta SF_{ay} & \delta SF_{az} \end{array}\right]$ is the scale factor error of the accelerometers, and $\delta SF_{g}=\left[\begin{array}{ccc} \delta SF_{gx} & \delta SF_{gy} & \delta SF_{gz} \end{array}\right]$ is the scale factor error of the gyroscopes. Unlike traditional tightly coupled integrated systems, CSAC provides precision time aiding. Clock bias and drift are not regarded as state variables.

Thus, the state equation can be written as:

(4) $$\dot{X}=A\cdot X+W$$

Based on error propagation function of MEMS INS in the navigation coordinate, A can be written in the Appendix.

### 3.2. Integration Observation Model

An observation model is described by the range or velocity error between satellites and the integration device. Suppose that the real position of the device is $R_{0}={\left[\begin{array}{ccc} x_{0} & y_{0} & z_{0} \end{array}\right]}^{T}$ and the calculated position of the device is $R_{\text{MINS}}={\left[\begin{array}{ccc} x_{\text{MINS}} & y_{\text{MINS}} & z_{\text{MINS}} \end{array}\right]}^{T}$, there are N visible satellites named “1,2⋯N” and the position of the satellite marked “n” is $R_{\left(n\right)}={\left[\begin{array}{ccc} x_{\left(n\right)} & y_{\left(n\right)} & z_{\left(n\right)} \end{array}\right]}^{T}$.

The position error is:

(5) $$\Delta R_{\text{MINS}}=R_{\text{MINS}}-R_{0}={\left[\begin{array}{ccc} x_{\text{MINS}}-x_{0} & y_{\text{MINS}}-y_{0} & z_{\text{MINS}}-z_{0} \end{array}\right]}^{T}$$

Thus,

(6) $$\begin{array}{l} \delta x=x_{\text{MINS}}-x_{0}\\ \delta y=y_{\text{MINS}}-y_{0}\\ \delta z=z_{\text{MINS}}-z_{0} \end{array}$$

The range between the device and the satellite n is derived based on calculated position.

(7) $$\rho_{\text{MINS}}^{\left(n\right)}=\left|R_{\left(n\right)}-R_{\text{MINS}}\right|=\sqrt{{\left(x_{\left(n\right)}-x_{\text{MINS}}\right)}^{2}+{\left(y_{\left(n\right)}-y_{\text{MINS}}\right)}^{2}+{\left(z_{\left(n\right)}-z_{\text{MINS}}\right)}^{2}}$$

Meanwhile, the real range is:

(8) $$r_{0}^{\left(n\right)}=\left|R_{\left(n\right)}-R_{0}\right|=\sqrt{{\left(x_{\left(n\right)}-x_{0}\right)}^{2}+{\left(y_{\left(n\right)}-y_{0}\right)}^{2}+{\left(z_{\left(n\right)}-z_{0}\right)}^{2}}$$

Taylor expansion of Equation (7) is deformed by neglecting higher order terms.

(9) $$\rho_{\text{MINS}}^{\left(n\right)}=r_{0}^{\left(n\right)}+\frac{x_{\left(n\right)}-x_{0}}{r_{0}^{\left(n\right)}}\delta x+\frac{y_{\left(n\right)}-y_{0}}{r_{0}^{\left(n\right)}}\delta y+\frac{z_{\left(n\right)}-z_{0}}{r_{0}^{\left(n\right)}}\delta z$$

CSAC clock coasting provides accurate time information in the integration. Thus, the GNSS receiver provides the range between the device and the satellite “n” as follows:

(10) $$\rho_{\text{GNSS}}^{\left(n\right)}=r_{0}^{\left(n\right)}+\Delta \rho_{c}^{\left(n\right)}+\varepsilon_{\delta t_{u}}$$

The range error of $\rho_{\text{GNSS}}^{\left(n\right)}$ can be divided into two parts (*i.e.*, $\Delta \rho_{c}^{\left(n\right)}$ and $\varepsilon_{\delta t_{u}}$). $\Delta \rho_{c}^{\left(n\right)}$ is the error described by factors except the receiver clock noise, which include the satellite clock offset, ionosphere delay, troposphere delay, ephemeris errors, and multipath error. $\varepsilon_{\delta t_{u}}$ is the pseudo range error caused by the receiver clock noise.

Hence,

(11) $$\Delta {\overset{⌢}{\rho }}_{c}^{\left(n\right)}=\Delta \rho_{c}^{\left(n\right)}+\varepsilon_{\delta t_{u}}$$

If N≥4, then CSAC aids positioning by weighted linear optimization. The covariance of $\Delta {\overset{⌢}{\rho }}_{c}$ is:

(12) $$\mathrm{cov}(\Delta {\overset{⌢}{\rho }}_{c})=\sigma_{\text{URE}}^{2}I+\sigma_{clock}^{2}OO^{T}$$

where $\sigma_{clock}^{2}$ is the receiver clock noise variance, and $\sigma_{\text{URE}}^{2}$ is the range error covariance of $\rho_{\text{GNSS}}^{\left(n\right)}$, which does not include the receiver clock error, I is a N×N unit matrix, and $O={\left[\begin{array}{cccc} 1 & 1 & \cdots & 1 \end{array}\right]}_{1\times N}^{T}$.

If N<4, then CSAC converts the GNSS observations to range measurements by “clock coasting”, which can constrain error divergence of micro inertial navigation, as well as improve the GNSS signals availability and positioning accuracy of the integration.

(13) $$\Delta \delta t_{u}=c\cdot (B_{f0}(t-t_{0})+\frac{1}{2}B_{f1}{\left(t-t_{0}\right)}^{2})$$

where c is the speed of light, $B_{f0}$ is the frequency bias of CSAC, $B_{f1}$ is the frequency drift of CSAC, and $t-t_{0}$ represents clock coasting time.

The range measurement error is:

(14) $$\begin{array}{ll} \delta \rho^{\left(n\right)} & =\rho_{\text{GNSS}}^{\left(n\right)}-\rho_{\text{MINS}}^{\left(n\right)}\\ & =-(\frac{x_{\left(n\right)}-x_{0}}{r_{0}^{\left(n\right)}}\delta x+\frac{y_{\left(n\right)}-y_{0}}{r_{0}^{\left(n\right)}}\delta y+\frac{z_{\left(n\right)}-z_{0}}{r_{0}^{\left(n\right)}}\delta z)+\Delta {\overset{⌢}{\rho }}_{c}^{\left(n\right)}\\ & =\left[\begin{array}{ccc} -\frac{x_{\left(n\right)}-x_{0}}{r_{0}^{\left(n\right)}} & -\frac{y_{\left(n\right)}-y_{0}}{r_{0}^{\left(n\right)}} & -\frac{z_{\left(n\right)}-z_{0}}{r_{0}^{\left(n\right)}} \end{array}\right]\left[\begin{array}{c} \delta x\\ \delta y\\ \delta z \end{array}\right]+\Delta {\overset{⌢}{\rho }}_{c}^{\left(n\right)} \end{array}$$

where

(15) $$\left[\begin{array}{c} \delta x\\ \delta y\\ \delta z \end{array}\right]=C_{\text{LLH}}^{\text{ECEF}}\left[\begin{array}{c} R\delta \varphi \\ R\cos\varphi \delta \lambda \\ \delta h \end{array}\right]=C_{\text{LLH}}^{\text{ECEF}}A_{12}^{-1}\left[\begin{array}{c} \delta \varphi \\ \delta \lambda \\ \delta h \end{array}\right]={\left(C_{\text{ECEF}}^{\text{LLH}}A_{12}\right)}^{-1}\left[\begin{array}{c} \delta \varphi \\ \delta \lambda \\ \delta h \end{array}\right]$$

(16) $$C_{\text{ECEF}}^{\text{LLH}}=\left[\begin{array}{ccc} -\sin\lambda & \cos\lambda & 0\\ -\sin\varphi \cos\lambda & -\sin\varphi \sin\lambda & \cos\varphi \\ \cos\varphi \cos\lambda & \cos\varphi \sin\lambda & \sin\varphi \end{array}\right]$$

$C_{\text{ECEF}}^{\text{LLH}}$ is the transform matrix from earth-centered earth-fixed (ECEF) coordinate system to site-centered coordinate system.

Hence,

(17) $$T_{\text{LLH}}^{\text{ECEF}}={\left(C_{\text{ECEF}}^{\text{LLH}}A_{12}\right)}^{-1}$$

When N satellites are visible, the range measurement error equation is written as:

(18) $$\delta \rho =M^{\prime }\left[\begin{array}{c} \delta x\\ \delta y\\ \delta z \end{array}\right]+\Delta \rho_{c}+\Delta \delta t_{u}\cdot O\;=M^{\prime }\cdot \left[\begin{array}{c} \delta x\\ \delta y\\ \delta z \end{array}\right]+\Delta {\overset{⌢}{\rho }}_{c}$$

where

(19) $$M^{\prime }=\left[\begin{array}{ccc} \frac{x_{0}-x_{\left(1\right)}}{r_{0}^{\left(1\right)}} & \frac{y_{0}-y_{\left(1\right)}}{r_{0}^{\left(1\right)}} & \frac{z_{0}-z_{\left(1\right)}}{r_{0}^{\left(1\right)}}\\ \frac{x_{0}-x_{\left(2\right)}}{r_{0}^{\left(2\right)}} & \frac{y_{0}-y_{\left(2\right)}}{r_{0}^{\left(2\right)}} & \frac{z_{0}-z_{\left(2\right)}}{r_{0}^{\left(2\right)}}\\ \vdots & \vdots & \vdots \\ \frac{x_{0}-x_{\left(N\right)}}{r_{0}^{\left(N\right)}} & \frac{y_{0}-y_{\left(N\right)}}{r_{0}^{\left(N\right)}} & \frac{z_{0}-z_{\left(N\right)}}{r_{0}^{\left(N\right)}} \end{array}\right]$$

The derivative of $r_{0}^{\left(n\right)}$ with time is:

(20) $${\dot{r}}_{0}^{\left(n\right)}=\frac{\left(x_{\left(n\right)}-x_{0})({\dot{x}}_{\left(n\right)}-{\dot{x}}_{0})+(y_{\left(n\right)}-y_{0})({\dot{y}}_{\left(n\right)}-{\dot{y}}_{0})+(z_{\left(n\right)}-z_{0})({\dot{z}}_{\left(n\right)}-{\dot{z}}_{0}\right)}{r_{0}^{\left(n\right)}}$$

The derivative of $\rho_{\text{MINS}}^{\left(n\right)}$ with time is:

(21) $$\begin{array}{ll} {\dot{\rho }}_{\text{MINS}}^{\left(n\right)} & ={\dot{r}}_{0}^{\left(n\right)}+\frac{\partial }{\partial t}(\frac{x_{\left(n\right)}-x_{0}}{r_{0}^{\left(n\right)}}\delta x+\frac{y_{\left(n\right)}-y_{0}}{r_{0}^{\left(n\right)}}\delta y+\frac{z_{\left(n\right)}-z_{0}}{r_{0}^{\left(n\right)}}\delta z)\\ & ={\dot{r}}_{0}^{\left(n\right)}+[\frac{{\dot{x}}_{\left(n\right)}-{\dot{x}}_{0}}{r_{0}^{\left(n\right)}}-\frac{(x_{\left(n\right)}-x_{0}){\dot{r}}_{0}^{\left(n\right)}}{{\left(r_{0}^{\left(n\right)}\right)}^{2}}]\delta x+[\frac{{\dot{y}}_{\left(n\right)}-{\dot{y}}_{0}}{r_{0}^{\left(n\right)}}-\frac{(y_{\left(n\right)}-y_{0}){\dot{r}}_{0}^{\left(n\right)}}{{\left(r_{0}^{\left(n\right)}\right)}^{2}}]\delta y\\ & \;+[\frac{{\dot{z}}_{\left(n\right)}-{\dot{z}}_{0}}{r_{0}^{\left(n\right)}}-\frac{(z_{\left(n\right)}-z_{0}){\dot{r}}_{0}^{\left(n\right)}}{{\left(r_{0}^{\left(n\right)}\right)}^{2}}]\delta z+\frac{x_{\left(n\right)}-x_{0}}{r_{0}^{\left(n\right)}}\delta \dot{x}+\frac{y_{\left(n\right)}-y_{0}}{r_{0}^{\left(n\right)}}\delta \dot{y}+\frac{z_{\left(n\right)}-z_{0}}{r_{0}^{\left(n\right)}}\delta \dot{z} \end{array}$$

The derivative of range between device and the satellite “n” from GNSS receiver with time is:

(22) $${\dot{\rho }}_{\text{GNSS}}^{\left(n\right)}={\dot{r}}_{0}^{\left(n\right)}+\xi^{\left(n\right)}$$

(23) $$\begin{array}{ll} \xi^{\left(n\right)} & =\xi_{Sat-Orbit}^{\left(n\right)}+\xi_{Sat-Clock}^{\left(n\right)}+\xi_{Sat-Speed}^{\left(n\right)}+\xi_{T+I}^{\left(n\right)}\\ & +\xi_{Rcv-Clock}^{\left(n\right)}+\xi_{Rcv-pos}^{\left(n\right)}+\xi_{Rcv-dopp}^{\left(n\right)} \end{array}$$

where,

$\xi_{Sat-Orbit}^{\left(n\right)}$ is the error of ${\dot{\rho }}_{\text{GNSS}}^{\left(n\right)}$ caused by orbit error, which is about 1 mm/s.

$\xi_{Sat-Clock}^{\left(n\right)}$ is the error of ${\dot{\rho }}_{\text{GNSS}}^{\left(n\right)}$ caused by clock drift, as the stability of atomic clock on the satellite is 10^−12^–10^−13^, which is approximately 0.3–0.03 mm/s and can be ignored.

$\xi_{Sat-Speed}^{\left(n\right)}$ is the error of ${\dot{\rho }}_{\text{GNSS}}^{\left(n\right)}$ caused by satellite velocity error, which is less than 1 mm/s.

$\xi_{T+I}^{\left(n\right)}$ is the error of ${\dot{\rho }}_{\text{GNSS}}^{\left(n\right)}$ caused by troposphere and ionosphere delays. Troposphere and ionosphere delays can be neglected.

$\xi_{Rcv-Clock}^{\left(n\right)}$ is the error of ${\dot{\rho }}_{\text{GNSS}}^{\left(n\right)}$ caused by receiver clock drift. The stability of CSAC is approximately 10^−11^. Thus, this error is 3–10 mm/s.

$\xi_{Rcv-pos}^{\left(n\right)}$ is the error of ${\dot{\rho }}_{\text{GNSS}}^{\left(n\right)}$ caused by receiver position error. This error is approximately 1.6 mm/s when the position error is 10 m.

$\xi_{Rcv-dopp}^{\left(n\right)}$ is the error of ${\dot{\rho }}_{\text{GNSS}}^{\left(n\right)}$ caused by receiver Doppler measurement error, which is approximately 0.7–1.4 mm/s.

The error of time derivative of range between device and satellite is measured by:

(24) $$\begin{array}{ll} \delta {\dot{\rho }}^{\left(n\right)} & ={\dot{\rho }}_{\text{GNSS}}^{\left(n\right)}-{\dot{\rho }}_{\text{MINS}}^{\left(n\right)}\\ & =\xi^{\left(n\right)}-\{[\frac{{\dot{x}}_{\left(n\right)}-{\dot{x}}_{0}}{r_{0}^{\left(n\right)}}-\frac{(x_{\left(n\right)}-x_{0}){\dot{r}}_{0}^{\left(n\right)}}{{\left(r_{0}^{\left(n\right)}\right)}^{2}}]\delta x+[\frac{{\dot{y}}_{\left(n\right)}-{\dot{y}}_{0}}{r_{0}^{\left(n\right)}}-\frac{(y_{\left(n\right)}-y_{0}){\dot{r}}_{0}^{\left(n\right)}}{{\left(r_{0}^{\left(n\right)}\right)}^{2}}]\delta y\\ & \;+[\frac{{\dot{z}}_{\left(n\right)}-{\dot{z}}_{0}}{r_{0}^{\left(n\right)}}-\frac{(z_{\left(n\right)}-z_{0}){\dot{r}}_{0}^{\left(n\right)}}{{\left(r_{0}^{\left(n\right)}\right)}^{2}}]\delta z+\frac{x_{\left(n\right)}-x_{0}}{r_{0}^{\left(n\right)}}\delta \dot{x}+\frac{y_{\left(n\right)}-y_{0}}{r_{0}^{\left(n\right)}}\delta \dot{y}+\frac{z_{\left(n\right)}-z_{0}}{r_{0}^{\left(n\right)}}\delta \dot{z}\} \end{array}$$

When the number of visible satellite is N, the time derivative of range error is written as follows:

(25) $$\delta \dot{\rho }=M^{\prime \prime }\left[\begin{array}{c} \delta x\\ \delta y\\ \delta z \end{array}\right]+M^{\prime }\left[\begin{array}{c} \delta \dot{x}\\ \delta \dot{y}\\ \delta \dot{z} \end{array}\right]+\xi$$

where

(26) $$M^{\prime \prime }=\left[\begin{array}{ccc} \frac{{\dot{x}}_{0}-{\dot{x}}_{\left(1\right)}}{r_{0}^{\left(1\right)}}-\frac{(x_{0}-x_{\left(1\right)}){\dot{r}}_{0}^{\left(1\right)}}{{\left(r_{0}^{\left(1\right)}\right)}^{2}} & \frac{{\dot{y}}_{0}-{\dot{y}}_{\left(1\right)}}{r_{0}^{\left(1\right)}}-\frac{(y_{0}-y_{\left(1\right)}){\dot{r}}_{0}^{\left(1\right)}}{{\left(r_{0}^{\left(1\right)}\right)}^{2}} & \frac{{\dot{z}}_{0}-{\dot{z}}_{\left(1\right)}}{r_{0}^{\left(1\right)}}-\frac{(z_{0}-z_{\left(1\right)}){\dot{r}}_{0}^{\left(1\right)}}{{\left(r_{0}^{\left(1\right)}\right)}^{2}}\\ \frac{{\dot{x}}_{0}-{\dot{x}}_{\left(2\right)}}{r_{0}^{\left(2\right)}}-\frac{(x_{0}-x_{\left(2\right)}){\dot{r}}_{0}^{\left(2\right)}}{{\left(r_{0}^{\left(2\right)}\right)}^{2}} & \frac{{\dot{y}}_{0}-{\dot{y}}_{\left(2\right)}}{r_{0}^{\left(2\right)}}-\frac{(y_{0}-y_{\left(2\right)}){\dot{r}}_{0}^{\left(2\right)}}{{\left(r_{0}^{\left(2\right)}\right)}^{2}} & \frac{{\dot{z}}_{0}-{\dot{z}}_{\left(2\right)}}{r_{0}^{\left(2\right)}}-\frac{(z_{0}-z_{\left(2\right)}){\dot{r}}_{0}^{\left(2\right)}}{{\left(r_{0}^{\left(2\right)}\right)}^{2}}\\ \vdots & \vdots & \vdots \\ \frac{{\dot{x}}_{0}-{\dot{x}}_{\left(N\right)}}{r_{0}^{\left(N\right)}}-\frac{(x_{0}-x_{\left(N\right)}){\dot{r}}_{0}^{\left(N\right)}}{{\left(r_{0}^{\left(N\right)}\right)}^{2}} & \frac{{\dot{y}}_{0}-{\dot{y}}_{\left(N\right)}}{r_{0}^{\left(N\right)}}-\frac{(y_{0}-y_{\left(N\right)}){\dot{r}}_{0}^{\left(N\right)}}{{\left(r_{0}^{\left(N\right)}\right)}^{2}} & \frac{{\dot{z}}_{0}-{\dot{z}}_{\left(N\right)}}{r_{0}^{\left(N\right)}}-\frac{(z_{0}-z_{\left(N\right)}){\dot{r}}_{0}^{\left(N\right)}}{{\left(r_{0}^{\left(N\right)}\right)}^{2}} \end{array}\right]$$

We then define the observation equation

(27) Z=C⋅X+U

Combining the range error and range rate error to measurement vector, we obtain:

(28) $$Z={\left[\begin{array}{c} \delta \rho \\ \delta \dot{\rho } \end{array}\right]}_{2N\times 1}$$

The observation matrix is:

(29) $$C={\left[\begin{array}{ccccccc} M^{\prime }\cdot T_{\text{LLH}}^{\text{ECEF}} & 0_{N\times 3} & 0_{N\times 3} & 0_{N\times 3} & 0_{N\times 3} & 0_{N\times 3} & 0_{N\times 3}\\ M^{\prime \prime }\cdot T_{\text{LLH}}^{\text{ECEF}} & M^{\prime }\cdot C_{\text{LLH}}^{\text{ECEF}} & 0_{N\times 3} & 0_{N\times 3} & 0_{N\times 3} & 0_{N\times 3} & 0_{N\times 3} \end{array}\right]}_{2N\times 21}$$

and the observation noise is:

(30) $$U={\left[\begin{array}{c} \Delta {\overset{⌢}{\rho }}_{c}\\ \xi \end{array}\right]}_{2N\times 1}$$

## 4. Field Vehicle Experiments

### 4.1. Experimental Setup

**Figure 2:**
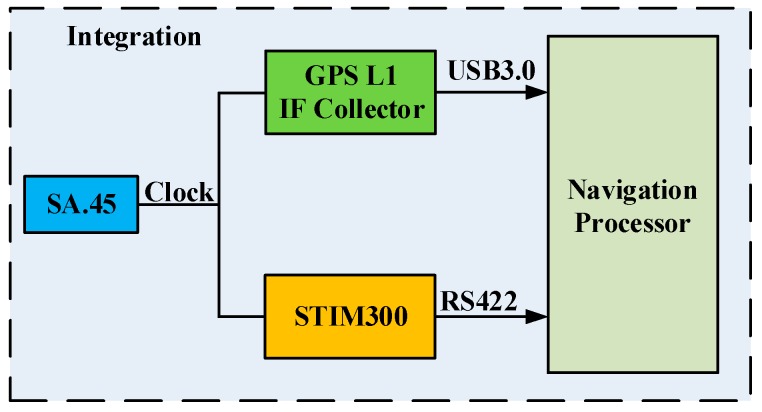
Hardware structure of the demo setup.

**Figure 3:**
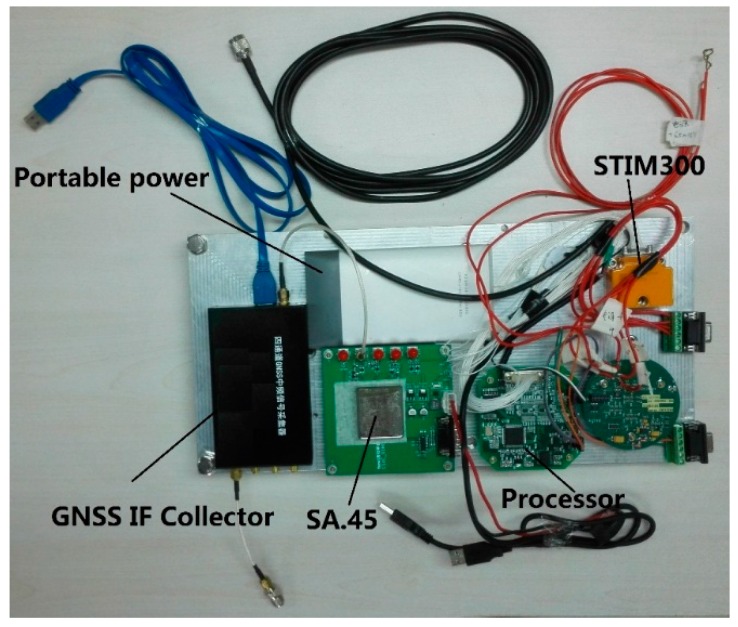
Photograph of the demo setup.

The demo setup of the coupled integration consisted of several parts: SA.45 CSAC, STIM300 MIMU, GPS L1 intermediate frequency signal collector, navigation processor, portable power, and several interfaces. The hardware structure of the demo setup is shown in Figure 2, and an actual photograph is shown in Figure 3. The main parameters of sensors in the demo setup are listed in Table 2.

**Figure 4:**
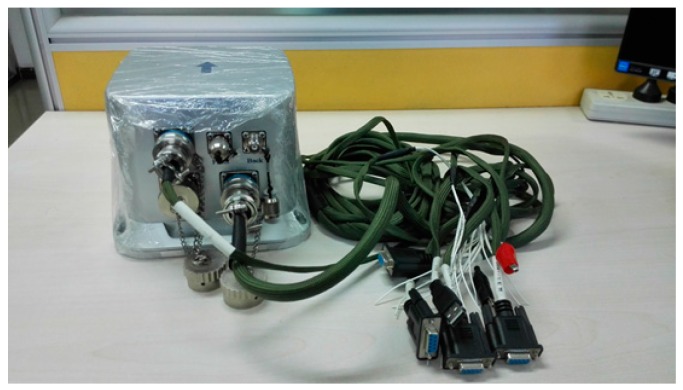
Photograph of GI7660.

An optical fiber combined with the inertial navigation system GI7660, which is manufactured by Beijing StarNeto Technology Development Co., Ltd. (Beijing, China), provided the reference trajectory. GI7660 consists of three close-loop optical fiber gyroscopes, three quartz accelerometers, and a mobile mapping grade multi-constellation multi-frequency GNSS receiver. The antenna of the GNSS receiver is HX-CS5601A, which is manufactured by Shenzhen Harxon Antenna Technology Co., Ltd. (Shenzhen, China). The main parameters of GI7660 are shown in Table 3, and an actual photograph is shown in Figure 4. The bias stability of GI7660 gyroscopes is 0.3°/h, whereas that of STIM300 is 6°/h with 10 s under average condition. The bias stability of GI7660 accelerometer is 20 μg, whereas that of STIM300 is 70 μg with 10 s under average conditions. Therefore, the inertial performance of GI7660 is much better than that of STIM300. In the tests, GI7660 worked in Real-Time Kinematic (RTK) mode.

The device for comparison consisted of a STIM300 and GPS L1 receiver driven by OCXO. The main parameters of OCXO are shown in Table 4.

**Figure 5:**
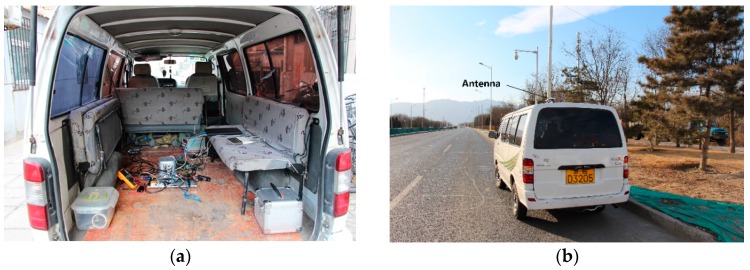
Photographs of the experimental vehicle. (**a**) Inner; (**b**) Exterior.

The demo setup, the reference device, and the compared setup were fixed in a vehicle, as shown in Figure 5, in which the same GNSS antenna is employed (*i.e.*, HX-CS5601A, Shenzhen Harxon Antenna Technology Co., Ltd., Shenzhen, China).

The navigation result refresh rate of the GI7660 is 10 Hz and was marked as “Ref”. The sample rate of the MIMU in the demo setup was 200 Hz. The inertial navigation result refresh rate of the MIMU with the two-sample iteration algorithm was 100 Hz and was marked as “CSAC+MIMU”. The positioning result refresh rate of the GPS L1 receiver in the demo setup was 1 Hz and was marked as “CSAC+GNSS”. The navigation result refresh rate of the demo with the integration algorithm aided by a precise clock was 100 Hz and was marked as “CSAC+MIMU+GNSS”. The navigation result refresh rate of the compared setup was 100 Hz and was marked as “MIMU+GNSS”.

### 4.2. Open-Sky Route

**Figure 6:**
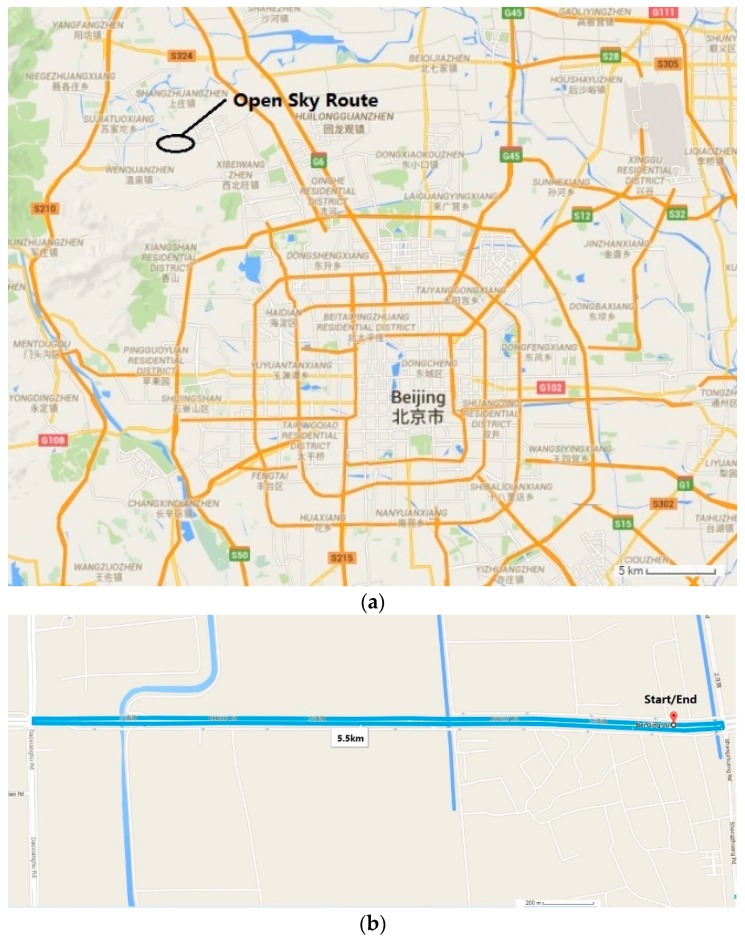
Map of the open-sky route (**a**) Schematic map; (**b**) Detail map.

Field vehicle experiments of open-sky route were conducted in Beiqing Road, Haidian District, Beijing, China. Figure 6 shows a map of the experiment route. The start point is marked by a red marker in the map (Figure 6b). The vehicle ran along the direction indicated by the black arrow and back to the start point. The journey was about 5.5 km. Before the experiments, MIMU was calibrated by the fast field calibration method. Then, the setup was fixed in the vehicle and kept stationary for 10 s for initial alignment. The vehicle started and ran along the predetermined route until it reached the terminal.

**Figure 7:**
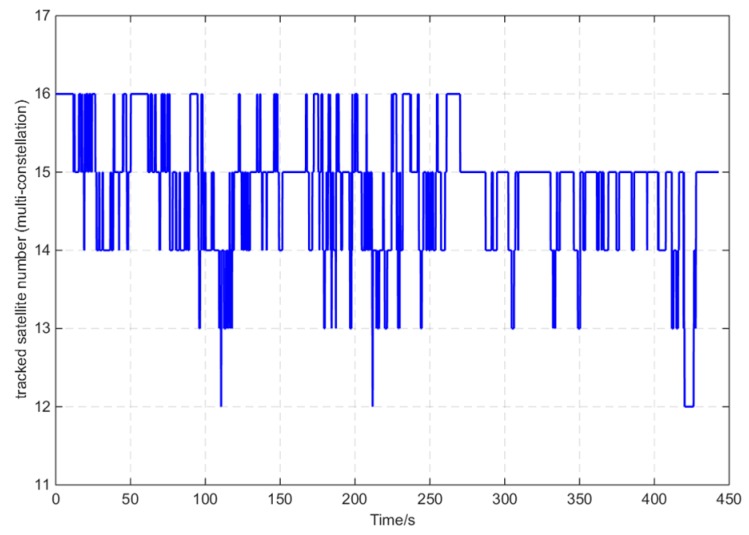
Tracked satellite number of GI7660.

**Figure 8:**
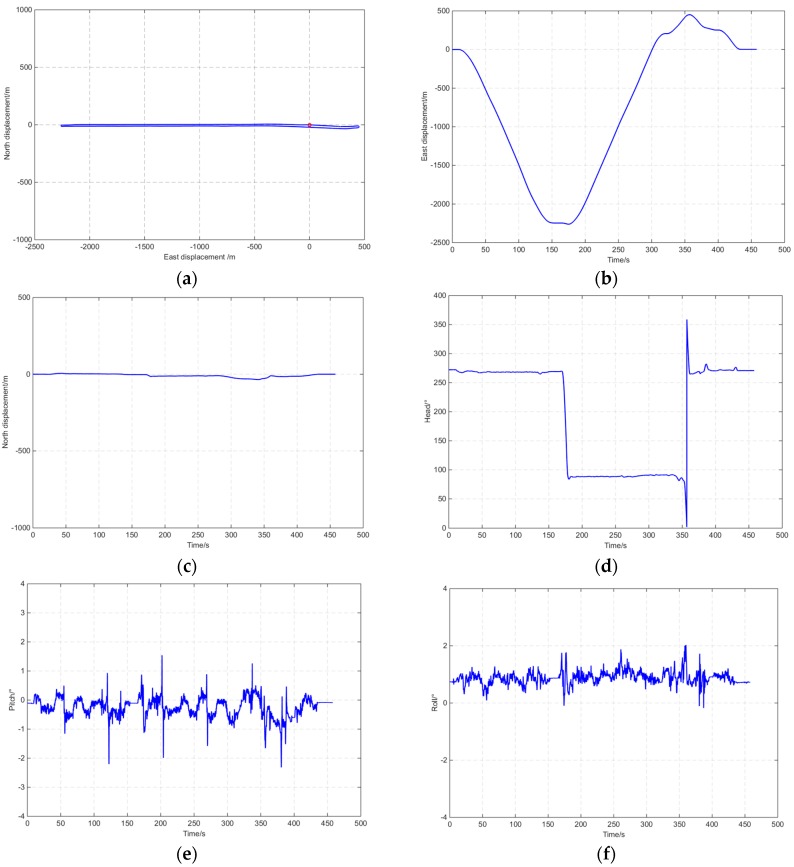
Reference route information measured by GI7660. (**a**) North and east displacement; (**b**) East displacement with time; (**c**) North displacement with time; (**d**) Head angle with time; (**e**) Pitch angle with time; (**f**) Roll angle with time.

The reference device GI7660 tracked the GPS (L1, L2, and L2C), BD2, GLONASS (L1 and L2), and Galileo (E1) constellation signals. Figure 7 shows the tracked satellite number of GI7660, and the average was 15. Figure 8 shows the reference information of the open-sky route measured by GI7660, including displacements and altitude with time.

**Figure 9:**
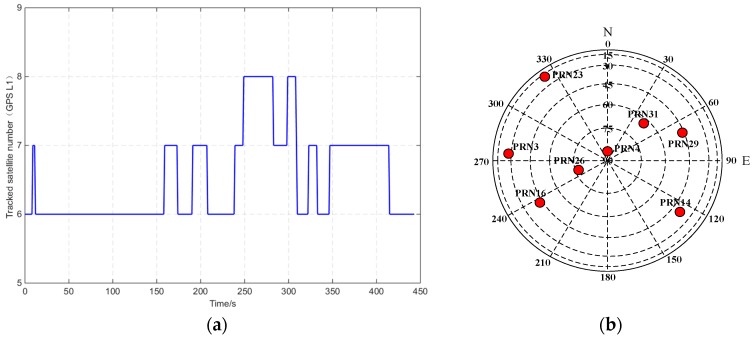
Tracked satellite number and space distribution of GPS L1 receiver. (**a**) Satellite number with time; (**b**) Space distribution.

Figure 9 shows the tracked satellite number and space distribution of the GPS L1 receiver in the demo and compared setups. The average number of tracked satellites was less than 7. PRN3, PRN4, PRN16, PRN26, PRN29, and PRN31 were tracked for the entire route.

**Figure 10:**
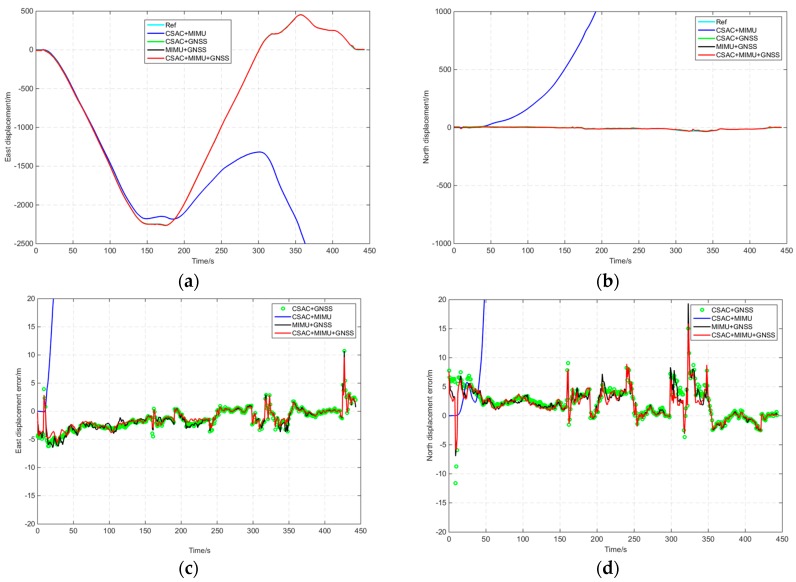
Navigation result comparison of the open-sky route. (**a**) East displacement with time; (**b**) North displacement with time; (**c**) East displacement error with time; (**d**) North displacement error with time.

The navigation results are compared in Figure 10 and Table 5. The result of the coupled integration navigation of the demo setup “CSAC+MIMU+GNSS” was better than that obtained by any other form. The horizontal positioning accuracy of “CSAC+MIMU+GNSS” was 13% higher than that of “CSAC+GNSS” and 21% higher than that of “MIMU+GNSS”.

**Figure 11:**
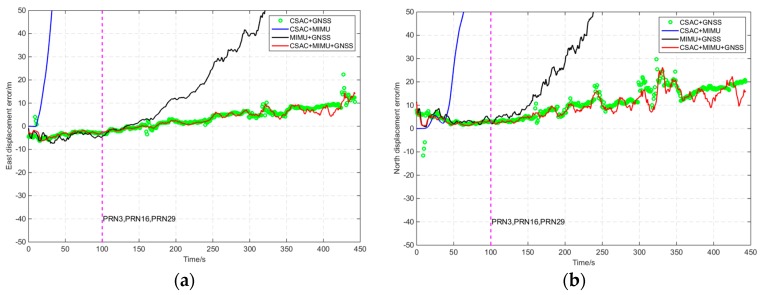
Navigation result comparison of the open-sky route by software mask. (**a**) East displacement error with time; (**b**) North displacement error with time.

A software mask was used to reduce the visible satellite number in the open-sky route. From 100 s to the final, only three satellites could be tracked because of the software mask. The visible satellites were PRN3, PRN16, and PRN29. The comparison of the navigation results by software mask is shown in Figure 11 and Table 6. After application of the software mask, “CSAC+GNSS” could still provide position information, and the positioning errors increased gradually with time. The positioning errors of “MIMU+GNSS” increased quickly with time, reaching >200 m at the end. The positioning errors of “CSAC+MIMU+GNSS” increased slowly and the horizontal error was 9.76 m, which was 14% more accurate than “CSAC+GNSS”.

### 4.3. Harsh Route

**Figure 12:**
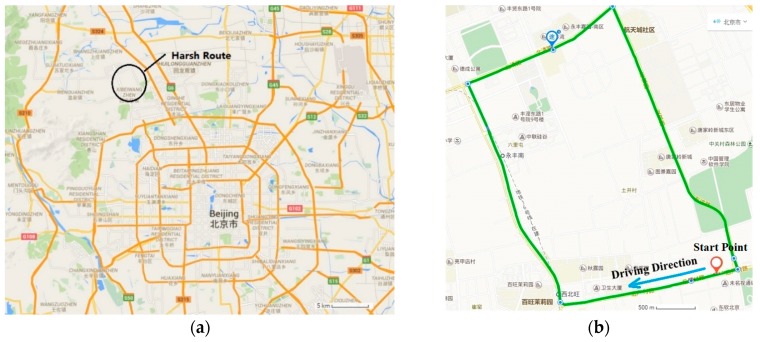
Map of harsh route (**a**) Schematic map; (**b**) Detail map.

**Figure 13:**
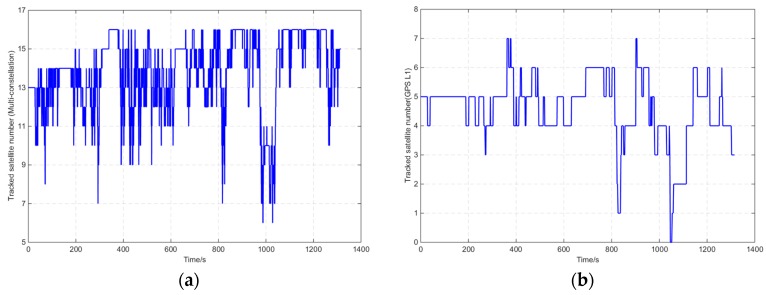
Number of tracked satellites for the harsh route. (**a**) GI7660; (**b**) Demo and compared setup.

Field experiments in harsh environments were conducted, and the route map is shown in Figure 12. The journey was about 11 km and lasted for approximately 1300 s. Figure 13 shows the number of tracked satellites with time for GI7660, the demo, and the compared setups. The average number for GI7660 was 12. The average number of the demo and compared setups was 4.6. At 1030–1113 s, severe occlusion occurred and less than three satellites could be tracked.

**Figure 14:**
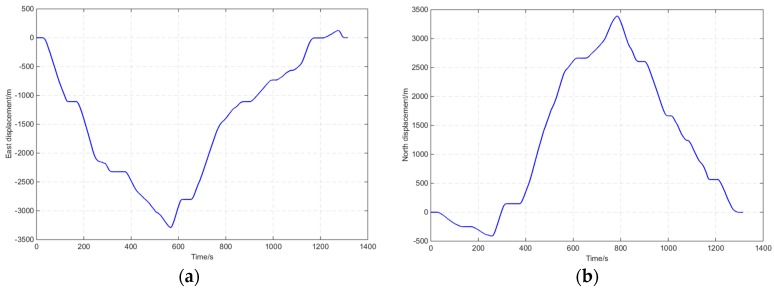

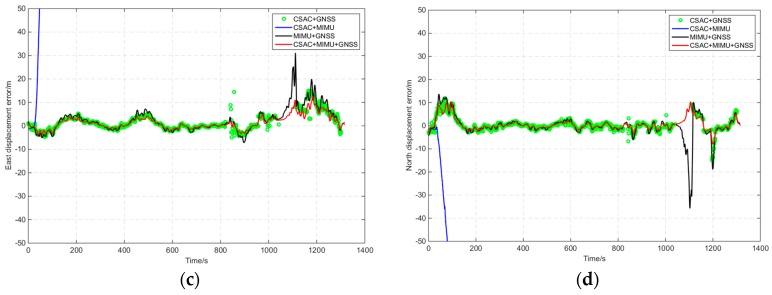
Displacements and errors for the harsh route. (**a**) East displacement with time of GI7660; (**b**) North displacement with time of GI7660; (**c**) East displacement error with time; (**d**) North displacement error with time.

Displacements and errors from the harsh route are shown in Figure 14, and navigation results are compared in Table 7. In Table 7 the statistical range in time domain of the “CSAC+GNSS” results was 89% of the whole route and the occlusion area is not included. The positioning probability of CSAC+GNSS was 89%. However, the statistical range in time domain of the “CSAC+MIMU+GNSS” results was 100% of the whole route and the occlusion area is included. The positioning error of the occlusion area expanded. This means that the statistical errors of “CSAC+MIMU+GNSS” are larger than “CSAC+GNSS”. The coupled integration positioning probability of the demo setup “CSAC+MIMU+GNSS” was 11% higher than that of “CSAC+GNSS”. The horizontal positioning accuracy of “CSAC+MIMU+GNSS” was 24% higher than that of “MIMU+GNSS”.

## 5. Conclusions

This paper discussed the coupled integration of CSAC, MIMU, and GNSS. The architecture of the integration was designed and the mathematical models of precise time aiding navigation filter were derived. If the visible satellite number was four or larger, then CSAC aided GNSS positioning with weighted linear optimization method and integrated with MIMU. If the visible satellite number was less than four, then CSAC converted GNSS observations to ranges by clock coasting and constrained the divergence of the MIMU inertial system. Field vehicle experiments were conducted for both open-sky and harsh routes. Results showed that the coupled integration was more accurate than the traditional techniques, including the tightly coupled GNSS/MIMU. Therefore, the coupled integration of CSAC, MIMU, and GNSS can improve PNT performance.

## Figures and Tables

**Table 1:** Degradations of the coupled integration.

Number	Subsystem Status			Mode
	CSAC	MIMU	GNSS	
1	√	√	√	Coupled integration
2	×	√	√	MIMU/GNSS tightly coupled integration
3	√	×	√	CSAC/GNSS coupled integration
4	√	√	×	Inertial navigation

**Table 2:** Main parameters of sensor in the setup.

Sensor	Parameter		Value
CSAC	Output		10 MHz (3.3V CMOS) 1 PPS
	Accuracy		<±5 × 10^−11^
	Short term frequency stability	@1 s	1.5 × 10^−10^
		@10 s	5 × 10^−11^
		@100 s	1.5 × 10^−11^
	Phase noise (dbc/Hz)	@1 Hz	<−55
		@10 Hz	<−78
		@100 Hz	<−113
		@1 KHz	<−128
		@10 KHz	<−135
	Power consumption		<120 mW
	Size		40.6 mm × 35.5 mm × 11.4 mm
MIMU	Gyroscope	Range	±400°/h
		Bias stability (Allan, 1σ)	0.5°/h
		Bias stability (Average time, 10 s)	6°/h
		Angle random walk	0.15°/√h
	Accelerometer	Range	±10 g
		Bias stability (Allan, 1σ)	50 μg
		Bias stability (Average time, 10 s)	70 μg
		Velocity random walk	0.06 m/s/√h
	Power consumption		1.5 W
	Weight		55 g
	Size		38.6 mm × 44.8 mm × 21.5 mm
GPS L1 IF collector	Chip type		MAX2769
	Signal		GPS L1
	Intermediate frequency		4.02 MHz
	Clock frequency		10 MHz
	Sample rate		20 MHz
	Sample digits		8 bit

**Table 3:** Main parameters of GI7660.

Parameter		Value	
Horizontal positioning accuracy	Single point positioning L1/L2		1.2 m (1σ)
	Differential Global Positioning System (DGPS)		0.4 m (1σ)
	RTK		2 cm (1σ)
Altitude accuracy			0.02°
Velocity accuracy			0.02 m/s (1σ)
Gyroscope	Type		Close-loop optical fiber
	Range		±300 °/s
	Stability (Average time, 10 s)		<0.3 °/h
Accelerometer	Type		Quartz
	Range		±10 g
	Stability (Average time, 10 s)		20 μg
Power consumption			20 W
Size		189 mm × 169 mm × 133 mm	

**Table 4:** Main parameters of OCXO.

Parameter		Value
Output frequency		10 MHz
Frequency stability		5 × 10^−8^
Phase noise	@10 Hz	<−95
	@100 Hz	<−125
	@1 KHz	<−135
	@10 KHz	<−150
Power consumption		3 W

**Table 5:** Navigation result comparison of the open-sky route.

Mode	East		North		Horizontal	
	Mean/m	Std/m	Mean/m	Std/m	Mean/m	Std/m
CSAC+GNSS	−1.44	1.95	2.25	2.66	3.50	2.40
CSAC+MIMU	>500		>500		>500	
MIMU+GNSS	−2.41	2.76	3.27	3.17	3.85	2.58
CSAC+MIMU+GNSS	−1.24	1.74	1.95	2.40	3.04	2.15

**Table 6:** Navigation result comparison of the open-sky route by software mask.

Mode	East		North		Horizontal	
	Mean/m	Std/m	Mean/m	Std/m	Mean/m	Std/m
CSAC+GNSS	2.50	4.91	10.07	6.41	11.38	6.59
CSAC+MIMU	>500		>500		>500	
MIMU+GNSS	>100		>100		>100	
CSAC+MIMU+GNSS	2.02	4.16	8.45	2.02	9.76	8.45

**Table 7:** Comparison of the navigation results for the harsh route.

Mode	East		North		Horizontal		Positioning Probability
	Mean/m	Std/m	Mean/m	Std/m	Mean/m	Std/m	
CSAC+GNSS	1.46	3.53	0.31	2.92	3.63	3.17	89%
CSAC+MIMU	>500		>500		>500		Failed
MIMU+GNSS	2.55	4.92	−1.18	5.10	5.37	5.43	100%
CSAC+MIMU+GNSS	1.83	3.89	0.95	3.59	4.10	3.79	100%

**Figure A1:**
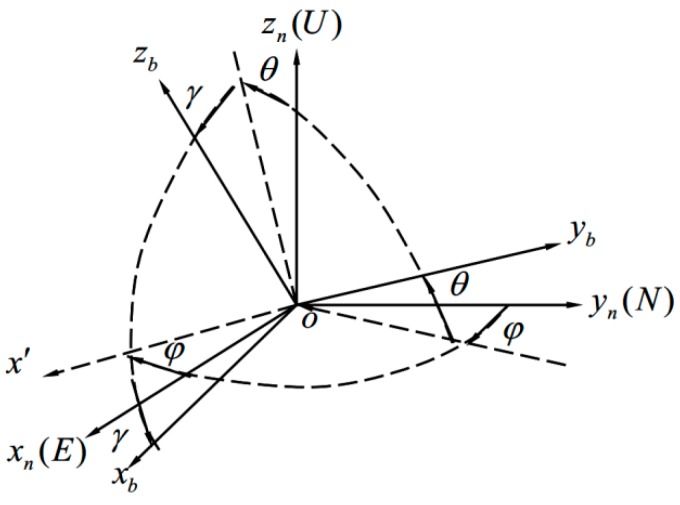
Carrier and navigation coordinate systems.

## Appendix A

The state matrix of the state equation can be written as:

(A1) $$Α=\left[\begin{array}{ccccccc} {Α}_{11} & {Α}_{12} & 0_{3\times 3} & 0_{3\times 3} & 0_{3\times 3} & 0_{3\times 3} & 0_{3\times 3}\\ {Α}_{21} & {Α}_{22} & {Α}_{23} & C_{b}^{n} & 0_{3\times 3} & C_{b}^{n}F^{b} & 0_{3\times 3}\\ {Α}_{31} & {Α}_{32} & {Α}_{33} & 0_{3\times 3} & C_{b}^{n} & 0_{3\times 3} & C_{b}^{n}\Omega^{b}\\ 0_{3\times 3} & 0_{3\times 3} & 0_{3\times 3} & {Α}_{44} & 0_{3\times 3} & 0_{3\times 3} & 0_{3\times 3}\\ 0_{3\times 3} & 0_{3\times 3} & 0_{3\times 3} & 0_{3\times 3} & {Α}_{55} & 0_{3\times 3} & 0_{3\times 3}\\ 0_{3\times 3} & 0_{3\times 3} & 0_{3\times 3} & 0_{3\times 3} & 0_{3\times 3} & {Α}_{66} & 0_{3\times 3}\\ 0_{3\times 3} & 0_{3\times 3} & 0_{3\times 3} & 0_{3\times 3} & 0_{3\times 3} & 0_{3\times 3} & {Α}_{77} \end{array}\right]$$

where

${Α}_{ij}$ is a 3 × 3 matrix,

(A2) $${Α}_{11}=\left[\begin{array}{ccc} 0 & 0 & -\frac{v_{N}}{R^{2}}\\ \frac{v_{E}\tan\varphi }{R\cos\varphi } & 0 & -\frac{v_{E}}{R^{2}\cos\varphi }\\ 0 & 0 & 0 \end{array}\right]$$

(A3) $${Α}_{12}=\left[\begin{array}{ccc} 0 & \frac{1}{R} & 0\\ \frac{1}{R\cos\varphi } & 0 & 0\\ 0 & 0 & 1 \end{array}\right]$$

(A4) $${Α}_{21}=\left[\begin{array}{ccc} 2\omega_{e}(v_{N}\cos\varphi -v_{D}\sin\varphi )+\frac{v_{N}v_{E}}{R\cos^{2}\varphi } & 0 & \frac{v_{E}}{R^{2}}(v_{U}-v_{N}\tan\varphi )\\ -v_{E}(2\omega_{e}\cos\varphi +\frac{v_{E}}{R\cos^{2}\varphi }) & 0 & \frac{1}{R^{2}}(v_{E}^{2}\tan\varphi +v_{E}v_{D})\\ -2\omega_{e}v_{E}\sin\varphi & 0 & -\frac{1}{R^{2}}(v_{E}^{2}+v_{N}^{2}) \end{array}\right]$$

(A5) $${Α}_{22}=\left[\begin{array}{ccc} \frac{1}{R}(v_{N}\tan\varphi -v_{U}) & 2\omega_{e}\sin\varphi +\frac{v_{E}\tan\varphi }{R} & -2\omega_{e}\cos\varphi -\frac{v_{E}}{R}\\ -2(\omega_{e}\sin\varphi +\frac{v_{E}\tan\varphi }{R}) & -\frac{v_{U}}{R} & -\frac{v_{N}}{R}\\ 2(\omega_{e}\cos\varphi +\frac{v_{E}}{R}) & \frac{2v_{N}}{R} & 0 \end{array}\right]$$

(A6) $${Α}_{23}=\left[\begin{array}{ccc} 0 & f_{U} & -f_{N}\\ -f_{U} & 0 & f_{E}\\ f_{N} & -f_{E} & 0 \end{array}\right]$$

$C_{b}^{n}$ is the transform matrix from carrier coordinate system $Ox_{b}y_{b}z_{b}$ to the navigation coordinate system $Ox_{n}y_{n}z_{n}$ in Figure A1. φ, θ, and γ are head, pitch, and roll angles, respectively.

(A7) $$F^{b}=diag(f^{b})=\left[\begin{array}{ccc} f_{x} & 0 & 0\\ 0 & f_{y} & 0\\ 0 & 0 & f_{z} \end{array}\right]$$

(A8) $$\Omega^{b}=diag(\omega^{b})=\left[\begin{array}{ccc} \omega_{x} & 0 & 0\\ 0 & \omega_{y} & 0\\ 0 & 0 & \omega_{z} \end{array}\right]$$

(A9) $${Α}_{31}=\left[\begin{array}{ccc} 0 & 0 & -\frac{v_{N}}{R^{2}}\\ \omega_{e}\sin\varphi & 0 & \frac{v_{E}}{R^{2}}\\ -\omega_{e}\cos\varphi -\frac{v_{E}}{R\cos^{2}\varphi } & 0 & \frac{v_{E}\tan\varphi }{R^{2}} \end{array}\right]$$

(A10) $${Α}_{32}=\left[\begin{array}{ccc} 0 & \frac{1}{R} & 0\\ -\frac{1}{R} & 0 & 0\\ -\frac{\tan\varphi }{R} & 0 & 0 \end{array}\right]$$

(A11) $${Α}_{33}=\left[\begin{array}{ccc} 0 & \omega_{e}\sin\varphi +\frac{v_{E}}{R}\tan\varphi & -(\omega_{e}\cos\varphi +\frac{v_{E}}{R})\\ -(\omega_{e}\sin\varphi +\frac{v_{E}}{R}\tan\varphi ) & 0 & -\frac{v_{N}}{R}\\ \omega_{e}\cos\varphi +\frac{v_{E}}{R} & \frac{v_{N}}{R} & 0 \end{array}\right]$$

(A12) $${Α}_{44}=\left[\begin{array}{ccc} -\tau_{\delta a}{}_{x} & 0 & 0\\ 0 & -\tau_{\delta ay} & 0\\ 0 & 0 & -\tau_{\delta az} \end{array}\right]$$

(A13) $${Α}_{55}=\left[\begin{array}{ccc} -\tau_{\delta gx} & 0 & 0\\ 0 & -\tau_{\delta gy} & 0\\ 0 & 0 & -\tau_{\delta gz} \end{array}\right]$$

(A14) $${Α}_{66}=\left[\begin{array}{ccc} -\tau_{\delta SFax} & 0 & 0\\ 0 & -\tau_{\delta SFay} & 0\\ 0 & 0 & -\tau_{\delta SFaz} \end{array}\right]$$

(A15) $${Α}_{77}=\left[\begin{array}{ccc} -\tau_{\delta SFgx} & 0 & 0\\ 0 & -\tau_{\delta SFgy} & 0\\ 0 & 0 & -\tau_{\delta SFgz} \end{array}\right]$$

$f_{E},f_{N},f_{U}$ are respectively the accelerations of east, north, and up directions in the navigation coordinate, $\tau_{\delta ax},\tau_{\delta ay},\tau_{\delta az}$ are the negative correlation coefficients of the three-axis accelerometer bias, $\tau_{\delta gx},\tau_{\delta gy},\tau_{\delta gz}$ are the negative correlation coefficients of the three-axis gyroscope bias, $\tau_{\delta SFax},\tau_{\delta SFay},\tau_{\delta SFaz}$ are the negative correlation coefficients of the three-axis accelerometer scale factor error, $\tau_{\delta SFgx},\tau_{\delta SFgy},\tau_{\delta SFgz}$ are the negative correlation coefficients of the three-axis gyroscope scale factor error, and R is the Earth’s radius.
